# Validation of Step Detection and Distance Calculation Algorithms for Soccer Performance Monitoring

**DOI:** 10.3390/s24113343

**Published:** 2024-05-23

**Authors:** Gabriele Santicchi, Susanna Stillavato, Marco Deriu, Aldo Comi, Pietro Cerveri, Fabio Esposito, Matteo Zago

**Affiliations:** 1 Department of Biomedical Sciences for Health, Università Statale di Milano, Via Mangiagalli 31, 20133 Milan, Italy; susanna.stillavato@unimi.it (S.S.); fabio.esposito@unimi.it (F.E.); matteo.zago@unimi.it (M.Z.); 2 Department of Electronics, Information and Bioengineering, Politecnico di Milano, Via Ponzio 34/5, 20133 Milan, Italy; marco1.deriu@mail.polimi.it (M.D.); pietro.cerveri@mail.polimi.it (P.C.); 3 Soccerment s.r.l, Viale Monza 259/265, 20126 Milan, Italy

**Keywords:** distance estimation, gyroscope-based algorithm, inertial measurement unit, soccer performance monitoring, step counter

## Abstract

This study focused on developing and evaluating a gyroscope-based step counter algorithm using inertial measurement unit (IMU) readings for precise athletic performance monitoring in soccer. The research aimed to provide reliable step detection and distance estimation tailored to soccer-specific movements, including various running speeds and directional changes. Real-time algorithms utilizing shank angular data from gyroscopes were created. Experiments were conducted on a specially designed soccer-specific testing circuit performed by 15 athletes, simulating a range of locomotion activities such as walking, jogging, and high-intensity actions. The algorithm outcome was compared with manually tagged data from a high-quality video camera-based system for validation, by assessing the agreement between the paired values using limits of agreement, concordance correlation coefficient, and further metrics. Results returned a step detection accuracy of 95.8% and a distance estimation Root Mean Square Error (RMSE) of 17.6 m over about 202 m of track. A sub-sample (*N* = 6) also wore two pairs of devices concurrently to evaluate inter-unit reliability. The performance analysis suggested that the algorithm was effective and reliable in tracking diverse soccer-specific movements. The proposed algorithm offered a robust and efficient solution for tracking step count and distance covered in soccer, particularly beneficial in indoor environments where global navigation satellite systems are not feasible. This advancement in sports technology widens the spectrum of tools for coaches and athletes in monitoring soccer performance.

## 1. Introduction

In recent years, the development of wearable devices has provided valuable tools for monitoring the athletic performance of soccer players during training and games. Metrics such as covered distance, distance within specific speed zones, accelerations, and decelerations have become essential for coaches, trainers, and sports scientists in evaluating player performance and preventing injuries. Many of these devices rely on global navigation satellite system (GNSS) sensors, with the Global Positioning System (GPS) being the most recognized system within this category. Their sampling rates, which depend on the manufacturer or the specific characteristics of the sensor itself, typically range from 5 Hz to 18 Hz [1,2,3,4].

However, satellite-based positional tracking sensors have limitations when applied to athletic performance monitoring. Firstly, studies have shown that GPS reliability in measuring the magnitude of high-intensity movements, such as accelerations and decelerations, is compromised [5,6,7,8]. Additionally, GPS devices are unsuitable for monitoring indoor environments due to multipath effects and signal attenuation caused by obstacles like walls [9,10,11]. This issue is particularly relevant in futsal, an indoor soccer-based sport recognized and promoted by FIFA and UEFA, which experienced a significant increase in participants and global recognition [12,13].

To address these challenges, inertial measurement units (IMUs) have been proposed as a prospective alternative [14,15]. These devices consist of miniaturized sensors containing accelerometers and gyroscopes, which measure linear acceleration and angular velocity, respectively. By integrating miniaturized IMUs into wearable devices, athletic data are gathered, and tailored algorithms can be applied to step detection and distance calculation [16], eliminating the need for additional equipment.

Previous research has focused on developing algorithms based on the placement of IMUs on various anatomical locations, including the trunk [17], wrist [18], hips [19], shank [20,21,22,23,24], and foot [25,26,27]. It has been observed that gait event detection is more precise when an IMU is worn on a lower limb, such as the shank or foot, rather than on the lower back [28,29]. Positioning the device on the shank, rather than on the foot, is advantageous for soccer, which involves frequent footwork including kicking and dribbling, as it not only reduces variability in IMU signals recorded across different subjects [21,30] but also helps avoid compromising the accuracy of step detection and movement analysis.

The algorithms developed for step detection and distance calculation typically utilize data from accelerometers [18,20,26,31], gyroscopes [21,22,23,32], or a combination of both [25,30], with significant differences in terms of required computational resources. A recent review of 17 algorithms developed for gait analysis demonstrated that gyroscope-based algorithms have superior performance in terms of accuracy and repeatability for gait event detection compared to accelerometer-based ones, particularly for detecting toe-off and heel-strike phases [25].

Recently, neural models and deep learning training techniques, renowned for their effectiveness in multidimensional signal processing and analysis, have been proposed for sequence-to-sequence learning synthesis and pattern recognition [33]. Architectures like Convolutional Neural Networks (CNNs) and Long Short-Term Memory networks (LSTMs) have proven adept at extracting complex patterns from IMU-generated time-series data [34,35]. This has led to their application in step detection [36,37,38,39,40] and stride length estimation [41]. Despite these advancements, deep learning models pose challenges, especially in real-time and resource-constrained environments typical of wearable devices.

It is noteworthy that existing algorithms have primarily been tested on straightforward walking and straight-line movements [29,37,42]. Their performance in diverse gait patterns, characteristic of soccer actions such as changes of direction (CoD), ball dribbling, and high-intensity activities remains largely unexplored. These complex movements disrupt the regular gait patterns, thereby challenging the applicability of existing algorithms in soccer-specific contexts.

This study seeks to address these existing limitations by meticulously developing real-time algorithms that utilize gyroscope data to accurately monitor key performance metrics for soccer players. These metrics include but are not limited to the total distance covered, the total number of steps taken, as well as distances covered at different intensity levels. Our work distinguishes itself through the specialized testing circuit designed to simulate a wide array of soccer-specific movements, such as jogging, sprinting, running with changes of direction, and ball dribbling. By doing so, this study aims to ensure that the developed algorithms are not just theoretically sound but also practically applicable and reliable in real-world soccer settings, from practice drills to actual matches.

The paper is structured as follows: After this introduction, Section 2 details the instrumentation, protocols, and algorithms integral to this study. Section 3 presents an in-depth analysis of the algorithm’s performance in various soccer-specific activities and paces, supported by a set of agreement metrics such as mean absolute percentage error (*MAPE*) and root mean squared error (*RMSE*). Additionally, metrics between video-tagged steps and algorithm-counted steps for each segment are obtained to validate the step detection accuracy. The inter-unit reliability of the algorithm is also assessed using a subset of participants who wore two devices simultaneously, allowing for the evaluation of consistency in the outputs. Section 4 serves as the discussion, offering an interpretative examination of the findings, exploring both the strengths and limitations of the algorithm, and discussing potential avenues for future improvements. Lastly, Section 5 concludes the paper by summarizing key findings and underscoring the algorithm’s significance in the advancement of athletic performance monitoring in soccer.

The distinct contributions of this research to the field include:

1. Development of a Real-Time, Gyroscope-Based Algorithm: tailored for low-resource environments, this algorithm is particularly suited to the constraints typical of wearable devices.
2. Introduction of an Innovative Testing Protocol: We have established a new protocol that covers a wide array of soccer movements. This innovation ensures that the practical applicability of the algorithm extends to soccer real-world scenarios.
3. Validation Against High-Definition Video Footage: to ensure enhanced accuracy and reliability, the algorithm has been rigorously tested against high-definition video footage for validating the step detection and distance estimation accuracy.

## 2. Materials and Methods

### 2.1. Hardware

The data collection process was performed using a pair of smart shin guards (XSEED, Soccerment, Milan, Italy) that contained GNSS sensors (sampling frequency: 10 Hz), and a 9-DoF IMU (sampling frequency: 200 Hz). The IMU includes a high dynamic accelerometer with a full-scale range (FSR) of up to ±64 g, a gyroscope with an FSR of up to ±2000 degrees per second, and a magnetometer with a dynamic range of ±50 Gauss. Each sensor communicates with a dual-core ARM ST32 microcontroller (STMicroelectronics, Geneva, Switzerland) either by I2C or SPI. The firmware version of the shin guards used in this study was 1.4.50. The suitability of the IMUs for accurate and repeatable gait parameter measurement has been well-documented [43]; indeed, these integrated sensors are both valid and reliable for measuring various spatiotemporal gait parameters such as gait speed, stance percent, swing percent, gait cycle time, stride length, cadence, and step duration [44].

**Figure 1:**
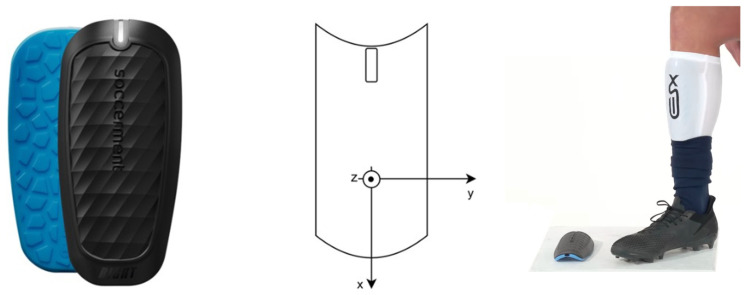
The XSEED smart shin guard and IMU Placement. (**Left**) The XSEED smart shin guard. (**Center**) Schematic of the shin guard showing the orientation of the IMU’s local reference system with axes labeled x,y, and z. (**Right**) Demonstration of the shin guard worn on the athlete’s leg, highlighting the placement of the device in situ.

The device (shown in Figure 1) analyzes continuously the data coming from the sensors and stores athletic and technical metrics in an embedded FLASH memory.

### 2.2. Participants and Data Acquisition

For this study, we recruited fifteen active and healthy male participants (*N* = 15; *mean* ± *SD*: age = 22.0 ± 4.1 years, height: 180.0 ± 6.5 cm, weight: 71.2 ± 6.3 kg) from the first team of an Italian semi-professional (Serie D, fourth division) soccer club. This selection aimed to evaluate the accuracy of the algorithms in real-world scenarios, reflecting the performance of skilled athletes. In compliance with ethical guidelines, all participants provided written informed consent. The study protocol was approved by the Ethics Committee of the Polytechnic University of Milan (id: 06/2020).

The experimental setup was meticulously designed to closely replicate typical soccer playing conditions. The trials were conducted outdoors on an artificial turf, chosen for its resemblance to common playing surfaces in competitive soccer. Participants were required to wear standard soccer shoes to ensure the authenticity of the soccer-related movements and to provide a consistent basis for data collection.

A carefully designed protocol, adapting the well-known circuit employed in FIFA quality performance assessment for electronic performance and tracking systems [45], was employed to collect gyroscope data that mimic various soccer-related paces. This included sprinting, walking, running with changes of direction (CoD), as well as ball dribbling. While maintaining the original circuit shape, we split straight-line and diagonal segments according to the type of pace to be analyzed. Participants wore a pair of XSEED shin guards throughout the testing, which was organized into time intervals for consistent performance duration across all subjects. Six participants wore a pair of devices on each leg throughout all sessions to assess the inter-unit reliability of the algorithm in distance estimation capabilities.

Following a 5-min personalized warm-up consisting of jogging and dynamic stretches, each participant completed five trials, each lasting 3 min, totaling a study duration of 15 min and covering an estimated distance of 1007.5 m. The length of each lap was measured to the nearest cm with a calibrated tape measure, and a goniometer was used to measure CoD sharpness. Poles and painted lines were used to guide the athletes and ensure adherence to the correct pathway, especially during the CoD segments. To synchronize the data captured across multiple devices, participants were instructed to perform three vertical jumps at the beginning of the data collection session. During periods of rest, participants were asked to remain stationary to prevent the recording of extraneous data. Data in which such conditions were not satisfactory have been excluded a priori before undergoing data processing and analysis.

**Figure 2:**
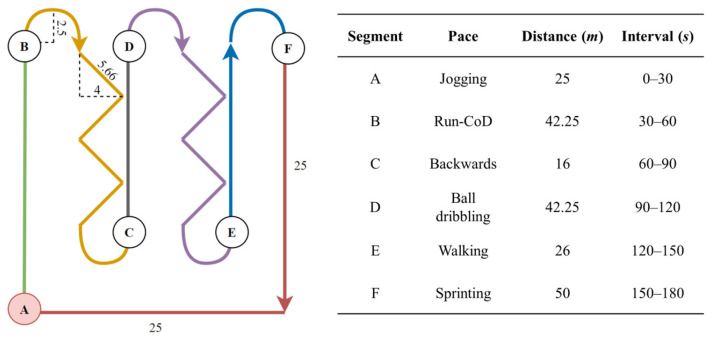
Track and distances covered during data acquisition. Each segment of the track is labeled with its corresponding pace, distance, and time interval.

The number of steps taken during each pace was manually labeled using a high-quality video camera-based system, allowing for precise identification and counting of steps by trained operators. The actual number of steps and distance covered in each segment served as a reliable benchmark for comparing the results obtained from the algorithms. Figure 2 illustrates the paths and time intervals of the track.

### 2.3. Data Preprocessing

**Figure 3:**
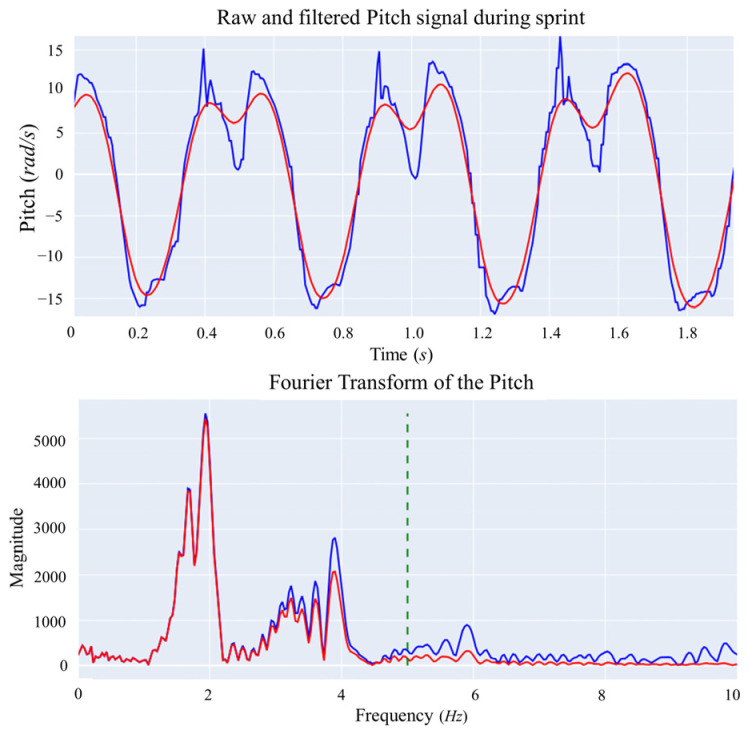
Filtering on the pitch signal. The top plot displays the raw (in blue) and filtered (in red) signals, and the bottom plot shows the Fourier Transform and the cut-off frequency of the Low-Pass Butterworth Filter. The dashed line refers to the cut-off frequency of the Low-Pass Filter applied.

The first step in the data preprocessing phase involved synchronizing the data streams from the two devices. This was achieved by aligning the data with the three vertical jumps performed by each athlete at the beginning of the session. Subsequent to this, a detailed analysis of the IMU readings was conducted, examining them in both the time and frequency domains. A Fourier Transform was applied to the gyroscope signals to identify and remove noisy components. Based on the characteristics of the signal and aligned with findings from previous research [22,46], a 2nd-order Butterworth filter with a 5 Hz cut-off frequency was applied to the raw signals. This filtering process, shown in Figure 3, was instrumental in preparing the data for further analysis related to step detection and distance calculation.

### 2.4. Gyroscope-Based Algorithm

The algorithm leverages the identification of gait events, which are primarily determined by observing the angular rate of the human shank, denoted as $\omega_{shank}$, along the sagittal plane (pitch axis). The human shank angular rate during gait activities has prominent peaks and troughs that correspond to toe off (*TO*), mid-swing (*MSW*), and initial contact (*IC*). Algorithms developed in previous works already leveraged these characteristics to perform gait step detection [21,23,47].

**Figure 4:**
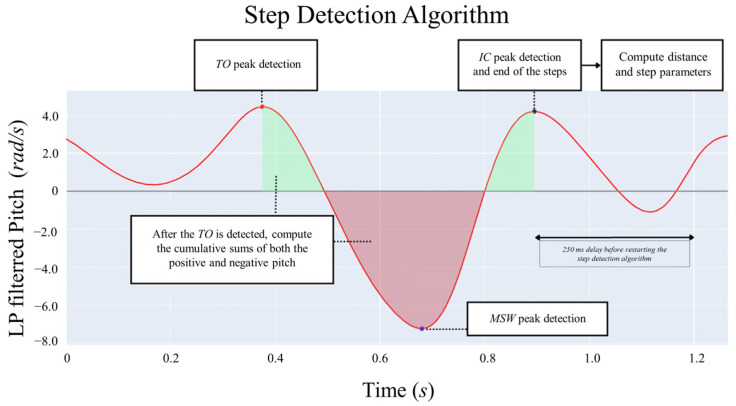
Step detection algorithm workflow. The peak detection is constrained to magnitude threshold and flight-time consistency that limit the number of false positive steps to be detected.

In the real-time algorithm workflow depicted in Figure 4, the process begins by searching for the maximum peak corresponding to *TO*, followed by detecting the *MSW* and completing the step detection upon identifying the *IC* peak. To enhance the accuracy of step detection and reduce the incidence of false positives, we have implemented specific thresholds in radians per second (rad/s) and flight-time consistency checks at each phase of the gait cycle. A magnitude threshold of 1 rad/s was chosen to accurately identify the *IC* and *TO* events. Furthermore, a temporal threshold was set at 250 ms based on the assumption that a maximum of 8 steps per second is a realistic expectation for soccer-specific movements, which precludes the algorithm from erroneously counting additional steps within this timeframe. When a backward step is detected, the algorithm adaptively inverts the sign of the peaks to be searched in subsequent steps, continuing this adjusted search until a new forward step is detected.

Upon the successful identification of these peaks, the algorithm then proceeds to compute the distance covered during each step. The distance calculation is based on the numerical integration of the gyroscope signal, which is directly proportional to the shank’s angular velocity. By multiplying the gyroscope values (in rad/s) by the time interval between consecutive samples (in seconds), the algorithm computes the angular displacement for each step.

The total angular displacement between the *TO* and *IC* events is determined by comparing the maximum value between positive and negative angular displacements of all samples within the step. This accounts for potential backward movements, as the displacement can be negative, and the algorithm can recognize such movements.

After each successful step detection, the gyroscope-driven data are reset. This helps mitigate the impact of drift phenomena resulting from the integration (and in turn propagation) of errors over longer durations [42].

**Figure 5:**
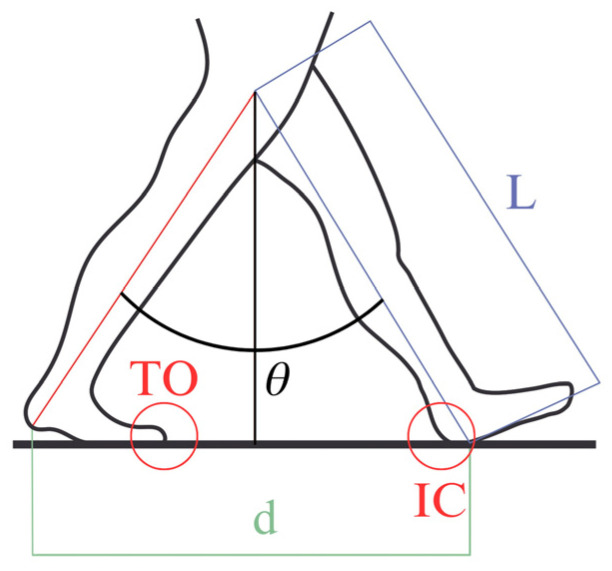
Trigonometric distance calculation.

The distance covered at each step was computed using trigonometric functions, as described in Figure 5 and in the following equation:

(1) $$d=L\;\cdot \sqrt{\left(2\left[1-\cos\left(\theta \right)\right]\right)}$$

where *L* is the length of the leg, and *θ* is the total angular displacement (in radians).

After the distance calculation, step parameters were saved into the FLASH memory of the device. To avoid multiple detections of the same step, the algorithm waits 250 ms before restarting from the search of the subsequent *TO*.

**Figure 6:**
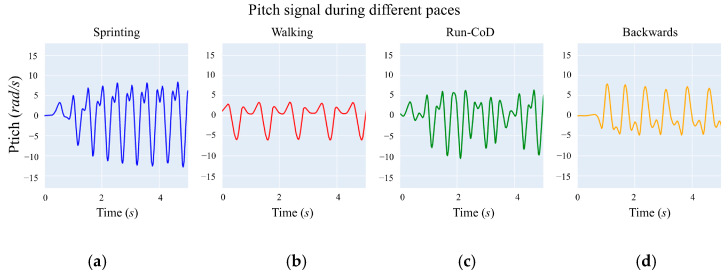
Comparison of the gyroscope signal across different soccer-specific movements. Note the high pitch variability during sprinting (**a**), which starts from a standing position, compared to walking (**b**). With changes in direction (**c**), the regularity of pitch is reduced, although the *TO*, *MSW*, and *IC* peaks are still observable. In the backward signal (**d**), the signs of these peaks are inverted, as expected.

Figure 6 illustrates the angular displacements occurring during different soccer-like movements such as walking, sprinting, running with CoD, and backward movements. In this curve, the minimum and maximum peaks represent the *TO*, *MSW*, and *IC* as previously described. The sprinting motion (Figure 6a) exhibits high variability in pitch compared to the walking pace (Figure 6b). Although the regularity of pitch in the run pace is reduced during CoD (Figure 6c), we observed that the *TO*, *MSW*, and *IC* peaks are still present. In backward movements (Figure 6d), the sign of these peaks is inverted, as expected.

### 2.5. Statistical Analysis

To evaluate the agreement between video-tagged steps and those estimated by the algorithm for each segment and athlete, we utilized a linear mixed model (LMM).

The LMM is expressed as:

(2) $$y_{ijlt}=\mu +\alpha_{i}+\beta_{j}+\gamma_{l}+{\left(\alpha \beta \right)}_{ij}+{\left(\alpha \gamma \right)}_{ij}+{\left(\beta \gamma \right)}_{jl}+\epsilon_{ijlt}$$

where $y_{ijlt}$ represents the steps measured by device j on subject i when performing activity l at time t; μ is the overall mean; $\alpha_{i}\sim N(0,\sigma_{\alpha }^{2})$ is the random subject effect; $\beta_{j}$ is the fixed effect of the device; $\gamma_{l}\sim N(0,\sigma_{\gamma }^{2})$ denotes the random activity effect; and $\epsilon_{ijlt}$ is the residual error. In this context, we initially include interaction terms to account for random interaction between athletes, activities, and devices. We assessed standard agreement model assumptions using Q-Q plots and standardized residuals, detailed in the Supplementary Materials. For analysis, we employed the lmer function from the R package lme4, which suits repeated measures data [48,49].

A systematic model comparison evaluated increasing complexity to determine the best balance between fit and parsimony, based on the Akaike information criterion (AIC) and Bayesian information criterion (BIC) [50]. Our final model, incorporating subject–device and subject–activity interactions, achieved the lowest AIC and BIC values, indicating optimal model complexity and fit.

Four statistical metrics were employed to assess the agreement between the step-counting algorithm and the video-tagged steps:

1. Concordance correlation coefficient (*CCC*), which assumes values ranging from −1 to +1, with +1 indicating perfect agreement between the paired values [51,52].
2. Limits of agreement (*LoA*), developed by Bland and Altman [51] to assess agreement between measures, have been adapted for repeated measures [53]. For *LoA* computation, a further LMM was fitted to “paired differences” denoting the between-device differences measured at the same time for each subject.
3. The total deviation index (*TDI*), which provides the boundary within which the differences will be contained p × 100% of the time, for a given containment probability p [54].
4. The coefficient of individual agreement (*CIA*), developed by Haber and Barnhart [55], which directly compares the disagreement between devices to the disagreement within devices within subjects. The value of the *CIA* ranges from 0 to 1, with 1 indicating that using different devices makes no difference to the variability of repeated measurements taken under the same conditions within the same subject.

These metrics together provide a robust framework for evaluating the algorithm. By using a diverse set of agreement indices, the analysis not only captures the magnitude of the biases and errors but also elucidates their sources.

The algorithm’s performance in estimating distances was evaluated in terms of mean absolute percentage error (*MAPE*), to provide a normalized measure of how the estimated distances deviate from the actual measurements, and root mean squared error (*RMSE*), to identify the magnitude of error in the estimations. In addition to the overall track analysis, a segment-wise evaluation was also conducted. This granular approach allows for the identification of any specific segments where the algorithm’s distance estimation might be less accurate, thus providing targeted areas for future improvements.

Finally, the assessment of inter-variability on athletes wearing two devices helped evaluate the consistency of paired measurements. In this assessment, the mean difference represents the average discrepancies between two devices, highlighting any overestimations or underestimations. The limits of agreement, comprising average lower and upper limits, delineate the range within which most differences fall. Lastly, the coefficient of variation (*CV*) quantifies the relative variation of the measurements, providing a gauge of their consistency.

## 3. Results

Fifteen athletes wore IMU and GNSS device shin guards, providing an average of 63 ± 5 paired measurements per pace. The total number of steps tagged and collected during this study was 13,202, distributed across various activities as follows: backward (1240 steps), ball dribbling (3586 steps), sprinting (2048 steps), walking (1978 steps), jogging (1571 steps), and run-CoD (2779 steps). Six participants were equipped with two units of the device on each leg to assess the inter-unit reliability of the results. The subsequent sections report and discuss the detailed outcomes related to step counting and distance estimation performances.

### 3.1. Step Detection

The linear mixed model (LMM) variance components (see Equation (2)) are as follows: $\sigma_{\alpha }^{2}=34.03,\;\sigma_{\gamma }^{2}=171.45,\;\sigma_{\alpha \gamma }^{2}=20.34,\;\sigma_{\alpha \beta }^{2}=0.65,$ and $\sigma_{\epsilon }^{2}=8.36$. The subject–device interaction and its terms $\sigma_{\beta \gamma }^{2}$ were excluded from the model upon an Akaike information criterion and Bayesian information criterion (AIC/BIC) evaluation on model complexity, which favored the model without such terms. The LMM variance components highlight that both the activity and subject identities are the principal sources of variability in our data. Notably, the subject–device interaction showed minimal impact, indicating negligible differential effects of the device across subjects.

The concordance correlation coefficient (*CCC*) was found to be 0.940, demonstrating excellent agreement between the estimated and actual step counts within the 95% confidence interval (*CI*) of 0.921 to 0.953. This high value suggests a robust concordance and confirms the reliability of the estimation method for accurately capturing step activity.

The total deviation index (*TDI*), which provides an upper limit on the absolute differences expected between the real and estimated steps 95% of the time, was calculated to be 9.57, with the bootstrap 95% *CI* of 8.61 to 10.47. This relatively narrow range underscores the precision of the estimation method, as it confines most deviations within a manageable magnitude, facilitating its practical application.

The coefficient of individual agreement (*CIA*), at 0.758 with a 95% *CI* of 0.636 to 0.841, further validates the high level of agreement on an individual basis, reinforcing the conclusion that the estimation method reliably approximates actual step counts across different subjects.

**Figure 7:**
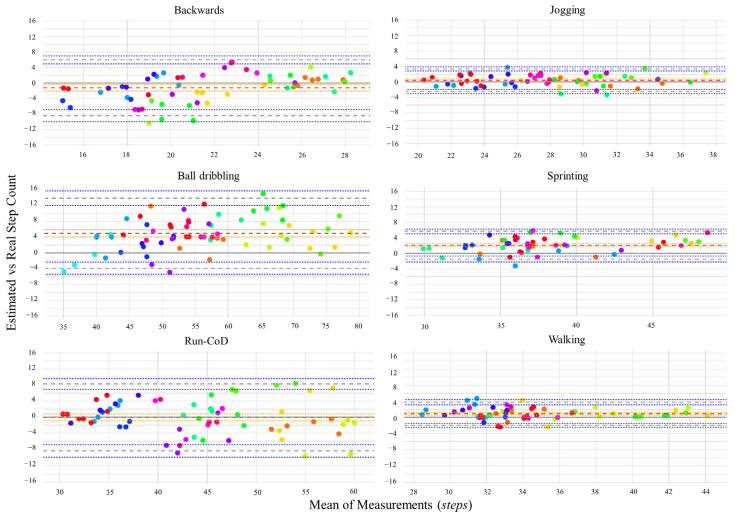
Bland–Altman plots grouped by pace. For each activity, the mean difference (red dashed line), as well as the lower and upper limits (blue dashed line), are reported with their 95% confidence intervals (dotted lines). Straight-line and regular activities are shown on the right, where the intervals are notably narrower. Each color represents repeated measures (laps) performed by individual athletes.

The limits of agreement (*LoA*), in the LMM modeling the differences, were found to have an overall average bias of 1.26 (95% *LoA* 0.77 to 1.68), with upper and lower bounds equal to 8.97 (95% *LoA* 7.84 to 10.91) and −6.44 (95% *LoA* −8.38 to −5.25), respectively. To assess the performance of the algorithm across different paces, *LoA* and *MAPE* were also computed for each activity. The results are detailed in Figure 7 and Table 1.

### 3.2. Distance Estimation and Inter-Unit Reliability

**Figure 8:**
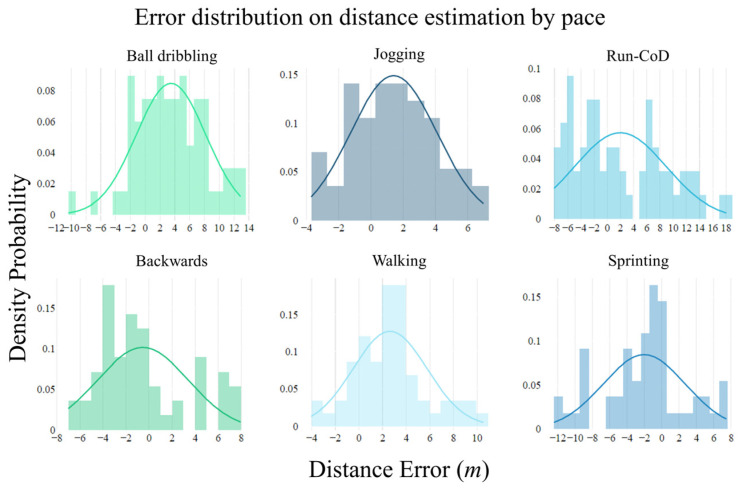
Distribution of distance estimation errors by activity type. The distribution’s density curve indicates the concentration of errors around the mean, providing insight into the algorithm’s precision in capturing actual distances across different movement patterns.

Figure 8 illustrates the error distribution in distance estimation by pace, which visually encapsulates the algorithm’s performance for different activities. Table 2 outlines the algorithm’s performance for each type of movement in terms of *mean* ± *SD*, *MAE*, *MAPE*, and *RMSE* values. For straight-line movements such as walking, jogging, and sprinting, the algorithm achieved accuracy levels of 88.7%, 91.0%, and 92.2%, respectively. The algorithm’s performance was less accurate for backward movements, with a recorded accuracy of 77.2%. In the case of ball dribbling and running with CoD, the algorithm accuracy drops to 88.2% and 85.9% accuracies. As depicted in Figure 8, errors in straight-line movements tend to cluster more tightly around zero, compared to errors in irregular activities. This suggests that the algorithm maintains a closer approximation to the actual distances during linear movements, with deviations more frequently converging toward the mean error of zero. This tendency signifies a more precise and consistent performance in straight-line paces relative to those involving more complex activities.

In addition to the evaluation of step detection performance, the inter-unit reliability of the algorithm for distance estimation was investigated. This assessment employed mean difference, limits of agreement (*LoA*), and coefficient of variation (*CV*). The mean difference between the outputs was close to zero (0.9 m), suggesting minimal bias between the units. The *LoA*, which defines the range of expected agreement between the units with 95% confidence, ranged from −2.03 m to 3.82 m. Finally, the *CV*, a measure of relative variability, was approximately 0.21, signifying a low level of variation between the distance estimates from the two units.

## 4. Discussion

The algorithm proposed to enable step count and distance measurement from a shin-mounted gyroscope demonstrated noticeable performance across a variety of soccer-relevant movements, with limited and documented inconsistencies in its accuracy under certain conditions.

Focusing on step detection, the algorithm leveraged the gyroscope readings to identify key gait events, namely toe off (*TO*), mid-swing (*MSW*), and initial contact (*IC*), based on prominent peaks and troughs in the angular rate of the human shank. Temporal and magnitude thresholds were applied during each phase of the real-time algorithm workflow to minimize false positive detections [30]. The algorithm achieved satisfactory accuracy rates for regular activities, such as walking, jogging, and straight-line sprinting, recording accuracies of 96.4%, 95.4%, and 93.6%, respectively. Compared to previous studies like Lee et al., who reported 99.1% accuracy using a shank-worn IMU for straight-line walking [31], and a review of 17 different algorithms applied to 420 strides [29], our results are in alignment, indicating a similar level of effectiveness. However, one of the key strengths of this study is the extensive dataset, which includes 13,202 manually tagged steps collected across a variety of soccer-specific activities. This large dataset enables a comprehensive evaluation of the algorithm’s performance under diverse and realistic conditions, distinguishing our work from previous studies that primarily focused on straight-line walking patterns.

For a detailed comparison of the performance metrics and computational requirements of these algorithms, please refer to Table 3.

Similarly to the study by Parker et al. [53], which investigated the agreement between a chest band and a gold-standard respiratory rate device, the current study provided a comprehensive set of agreement metrics to evaluate the performance of the step-counting algorithm with respect to a steps video tagging. These metrics, reported in Table 4, provided a more nuanced understanding of how well the estimated step counts corresponded to the actual steps, moving beyond a simple focus on accuracy percentages. The concordance correlation coefficient (*CCC*) achieved a value of 0.940 (with a narrow confidence interval), signifying excellent agreement between the estimated and actual steps. This reinforces the algorithm’s reliability in capturing true step activity.

The total deviation index (*TDI*) provided further evidence for the algorithm’s precision. The relatively narrow value of 9.569 suggested a limited range of deviations between estimated and actual steps. This ensured that most estimations were within a manageable margin of error. The coefficient of individual agreement (*CIA*) complemented the other metrics by emphasizing agreement on an individual basis. The high *CIA* value of 0.758 (with a confidence interval) underscored a strong level of agreement, implying that the algorithm performed consistently across different subjects regardless of variations in gait patterns.

The limits of agreement indicated a modest overall average bias of 1.26, with 95% confidence ranging from 0.77 to 1.68. The upper and lower bounds were calculated at 8.97 (95% confidence from 7.84 to 10.91) and −6.44 (95% confidence from −8.38 to −5.25), respectively, signifying that deviations from actual step counts were generally small. This array of agreement metrics provided a robust assessment that went beyond mere accuracy percentages, offering a nuanced appraisal that bolsters confidence in the algorithm’s capability for precise real-time step detection and distance estimation.

Built upon the gyroscope-based step detection mechanism, the algorithm utilized numerical integration and trigonometric functions to estimate distance. This was calculated based on the angular displacement between the toe off and initial contact events during each step, incorporating a mathematical model that also factored in the length of the leg. Although the core formula (Equation (1)) was adapted from the existing literature [42], the angle displacement computation (see Section 2.4) was designed to be applicable to a wider range of paces beyond just regular walking, which was the focus of previous studies.

The algorithm’s performance was highly accurate for a variety of movements. The algorithm demonstrated notable accuracy in high-intensity activities like sprinting. Despite a slight underestimation (average estimated distance: 48.4 m vs. reference: 50 m), the root mean squared error (*RMSE*) was 5.2 m over a 50 m distance. This highlights the algorithm’s ability to adapt to fast movements crucial in soccer and can be attributed to the precise identification of angular displacements and the subsequent trigonometric calculations, as shown in Equation (1).

In contrast, low-intensity activities like walking (estimated distance: 28.4 m vs. reference: 26 m) and jogging (estimated distance: 44.5 m vs. reference: 42.3 m) exhibited a tendency for overestimation. This overestimation is likely attributable to the use of a low-order low-pass filter applied to the gyroscope signal. While this filter serves to smooth the signal, its lower order may inadvertently reduce peak smoothing, leading to an overestimation of the angular displacement. High-order filters, which could mitigate this effect, were not employed to avoid distorting the signal during high-intensity movements, which are crucial for assessing soccer performance.

The algorithm faced challenges with backward movement, achieving an *RMSE* of 4.2 over a 16 m distance. This result can be partly attributed to the challenges associated with recognizing backward steps based on gyroscope data. The algorithm detects such steps by comparing the positive and negative angular displacements, then, it takes the bigger one and then computes the distance using the absolute value of it. Although our approach allows the algorithm to detect both forward and backward movements by comparing the magnitudes of positive and negative angular displacements, it appears that further refinements are needed to bolster its robustness and reliability.

Moreover, the inter-unit reliability analysis showed a high degree of consistency between two devices worn on the same leg for distance estimation. The slight mean difference (0.9) and the tight limits of agreement (*LoA* ranging from −2.03 to 3.80) reinforce this conclusion. A low coefficient of variation (*CV* = 0.21) also indicates minimal variability between the units, underscoring their consistent distance estimation. This consistency is essential when comparing performance across different athletes, confirming the algorithm’s reliability when used with multiple sensor units.

In essence, the distance estimation algorithm showcased strong potential in most scenarios, with a few areas where further refinement might be beneficial. This can be attributed to the lower accuracy in the step detection and the signal filtering processes. Notably, the estimation accuracy varied across different paces; while in low-intensity movements such as walking and jogging, distance overestimation was observed, in high-intensity movements like running and sprinting, a slight distance underestimation was reported.

Despite its simplicity, our gyroscope-based algorithm excels in resource efficiency, crucial for wearable devices. This stands in contrast to deep learning models which, despite their advanced capabilities, face challenges in the resource-limited environments of wearables. These models require extensive datasets, high computational power, and often need offline preprocessing, not feasible for real-time applications in embedded systems [27,35,38]. Our algorithm’s effectiveness within these constraints highlights its practicality for real-time athletic performance monitoring.

**Figure 9:**
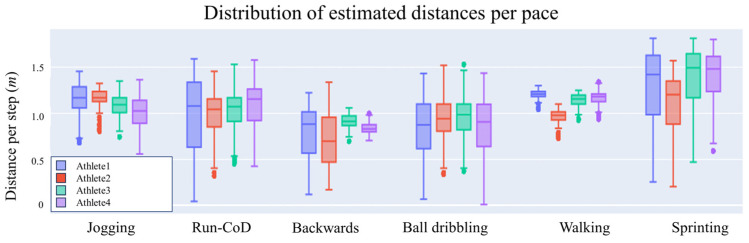
Estimated distance covered in each step at different paces. The colors allow for easy comparison between athletes within each running pace.

Figure 9 illustrates the distribution of distances in each step for different paces. The boxplots are categorized by pace and corresponding athlete. As expected, the distances calculated during walking and jogging activities, characterized by their regular patterns, exhibit low variability throughout the entire session. Conversely, running with CoD, ball dribbling, and sprinting activities demonstrate considerable variability. In particular, the sprinting activity is characterized by an increase in the distance covered during accelerations, followed by a decrease in the last few meters.

### 4.1. Limitations

While this study successfully assessed the algorithm’s performance using agreement metrics, some limitations require attention. Firstly, the relatively narrow range of participants’ body size might restrict the generalizability of the results to a more varied population with different gait patterns. Secondly, the impact of variable sensor placement on shin guards, which can differ in actual play, on the accuracy of measurements was not considered. Lastly, the controlled nature of the trial setting may not accurately represent the dynamic conditions of an actual soccer game, including abrupt turns, varied playing surfaces, and player interactions. To enhance the algorithm’s applicability, future studies should extend the research to include these factors, thereby validating its efficacy in more diverse and realistic environments.

### 4.2. Future Improvements

Future research will also focus on a more detailed analysis of step detection errors, a critical factor in the accuracy of distance estimation. This analysis will specifically target the identification of primary error sources, such as missed steps, particularly in backward movements, signal noise, and abrupt changes in movement patterns. Understanding and addressing these sources of errors is crucial for enhancing the robustness and reliability of the algorithm.

Further efforts will be devoted to expanding the dataset size to include a more diverse range of participants and the assessment in training or official matches. This expansion is expected to provide a more comprehensive understanding of the algorithm’s performance across varied scenarios and under different conditions, such as fatigue.

Additionally, we aim to validate the algorithm’s estimated instantaneous speed against a gold-standard reference, such as a laser gun, which is vital for developing advanced athletic metrics encompassing not just distance but also speed, acceleration, and deceleration. The integration of accelerometer data with our current gyroscope-based algorithm is anticipated to offer enhanced accuracy and insights into step pattern imbalances, potentially aiding in injury prevention. Merging gyroscope data with GPS inputs from the same wearable device could provide a more comprehensive analysis of player movements, combining both macro- and micro-level data for a holistic performance assessment. These improvements would not only refine the algorithm’s accuracy but also expand its practical adoption in professional soccer analytics.

## 5. Conclusions

In this study, we have developed and validated a gyroscope-based algorithm for step detection and distance estimation tailored for IMU-equipped shin guards. The algorithm notched a step detection accuracy of 95.8% and an RMSE of 17.6 m over a 202 m track, proving its efficacy for a range of soccer-related movements. It adeptly captures both forward and backward steps, enhancing its utility for detailed athletic motion analysis, though some limitations were observed. The algorithm’s robustness was carefully examined with a suite of agreement metrics that confirmed its reliability and consistency across devices. With its precision, real-time operational capacity, and relevance to soccer, the algorithm stands as a practical tool for athletes, coaches, and sports scientists. Overall, this algorithm is a valuable contribution to the resources for advancing athletic performance analysis in the field of soccer.

## Figures and Tables

**Table 1:** Step detection accuracy. The 95% confidence interval (*CI*) is reported in round brackets.

Pace	Real Steps *mean* ± *SD*	Estimated *mean* ± *SD*	Mean Difference	Lower Limit	Upper Limit	*MAPE* (%)
Jogging	27.6 ± 4.2	28.0 ± 4.3	0.4 (0.1; 0.8)	−2.5 (−3.0; −1.9)	3.3 (2.8; 3.9)	4.6
Run-CoD	44.1 ± 9.2	44.0 ± 8.6	−0.1 (−0.9; 0.8)	−8.4 (−9.9; −6.8)	8.2 (6.9; 9.5)	7.4
Backward	22.1 ± 3.2	20.9 ± 4.8	−1.3 (−2.0; −0.4)	−8.5 (−10.1; −6.8)	6.0 (5.1; 7.0)	14.0
Ball Dribbling	53.5 ± 10.2	58.5 ± 11.5	5.0 (4.0; 5.9)	−3.9 (−5.4; −2.4)	13.8 (12.1; 15.5)	10.7
Walking	34.1 ± 3.9	35.0 ± 3.7	0.9 (0.6; 1.2)	−2.0 (−2.4; −1.5)	3.7 (3.1; 4.3)	3.6
Sprinting	37.2 ± 4.5	39.3 ± 5.0	2.1 (1.7; 2.5)	−1.5 (−2.3; −0.8)	5.7 (5.1; 6.3)	6.4
Total	217.2 ± 26.2	224.5 ± 26.0	1.3 (0.8; 1.7)	−6.4 (−8.4; −5.2)	9.0 (7.8; 10.9)	4.2

**Table 2:** Distance estimation accuracy.

Pace	Reference (m)	Estimated (*mean* ± *std*)	*MAE* (m)	*RMSE* (m)	*MAPE* (%)
Jogging	25.0	26.4 ± 2.4	2.3	2.7	9.0
Run-CoD	42.3	44.5 ± 7.3	5.9	7.5	14.1
Backward	16.0	15.3 ± 4.2	3.7	4.2	22.8
Ball dribbling	42.3	44.5 ± 7.3	5.0	6.1	11.8
Walking	26.0	28.4 ± 3.0	2.9	3.8	11.3
Sprinting	50.0	48.4 ± 4.9	3.9	5.2	7.8
Total	201.6	209.6 ± 15.8	14.2	17.6	7.0

**Table 3:** Summary of previous state-of-the-art methods. Accuracy is reported in MAPE (%). N/A indicates that data for the specified activity were not collected in the study.

Algorithm	Dataset	Input Data	Step Detection Accuracy		
			Walking	Running	Others *
Proposed (Current Study)	13,202 steps from 15 subjects, walking, jogging, sprinting, and various soccer-related movements *.	Single-axis gyroscope measurements from shanks; positive and negative angular displacement for backward	96.4%	95.4%	86–93.6%
Khandelwal & Wickström (2017) [56]	20 subjects, various activities: walking, indoor/outdoor running, treadmill	3-axis accelerometer data placed on waist, wrist, and ankles	F1 score: 0.94–0.98	F1 score: 0.53–0.82	N/A
Romijnders et al. (2022) [28]	157 participants, straight-line walking, controlled indoor environment	3-axis accelerometer and gyroscope data from shank	F1 score 0.94–0.96	N/A	N/A
Bertuletti et al. (2018) [57]	5077 steps during rectilinear and curvilinear walking by 16 healthy adults	3-axis accelerometer and gyroscope data, two time-of-flight distance sensors on feet	88.8–99.8%	N/A	N/A
Lee et al. (2010) [31]	10 subjects, straight-line walking at variable speeds	3-axis accelerometer data from ankles and footswitches	99.1%	N/A	N/A
Catalfamo et al. (2010) [21]	455 steps during walking on level ground and inclines	Single-axis gyroscope measurements from shank.	98.9%	N/A	N/A

* Note: “Others” refers to various soccer-related movements such as backward walking, running with CoD, and ball dribbling.

**Table 4:** Summary of agreement metrics between estimated and actual step counts.

Agreement Metric	Values and Confidence Interval	Interpretation
Concordance Correlation Coefficient	0.94 (95% CI: 0.92 to 0.95)	Demonstrated excellent agreement between the estimated and actual step counts, confirming the algorithm’s reliability.
Total Deviation Index	9.57 (95% CI: 8.62 to 10.47)	Provides an upper limit on absolute differences, underscoring the precision of the estimation method.
Coefficient of Individual Agreement	0.758 (95% CI: 0.64 to 0.84)	Validates high individual agreement, ensuring the method’s consistency across different subjects.
Limits of Agreement	Average Bias: 1.3 (95% LoA: 0.8 to 1.7)	Shows the algorithm’s accuracy with upper and lower bounds defined, indicating minimal bias in measurements.

## Data Availability

The dataset used in this study is not sharable due to privacy and ethical restrictions. The data were provided by a private company, and access is limited to the researchers involved in this study in compliance with confidentiality agreements.

## Supplementary Materials

The following supporting information can be downloaded at: https://www.mdpi.com/article/10.3390/s24113343/s1: Figure S1: Q-Q plot of raw conditional residuals (S1_raw_conditional_res_qqplot). This figure assesses the normality of the residuals from the linear mixed model (LMM) used in the statistical analysis. Figure S2: Plot of raw conditional residuals and Figure S3: Plot of Pearson conditional residuals. These figures show the distribution of the raw and Pearson residuals against the fitted values, indicating that no patterns are present, further suggesting model adequacy. Figure S4: Comparison of AIC and BIC values for different LMMs. This figure illustrates the Akaike Information Criterion (AIC) and Bayesian Information Criterion (BIC) values for various models to determine the best fit. The best fit was obtained with model complexity 3, in which the subject-device interaction terms were excluded.
