# Supplementary material for: Validation of Step Detection and Distance Calculation Algorithms for Soccer Performance Monitoring
Source: Sensors (Basel). 2024 May 23;24(11):3343. doi: 10.3390/s24113343 (PMC11174549; doi:10.3390/s24113343)

Figure S4: Comparison of AIC and BIC values for different LMMs.

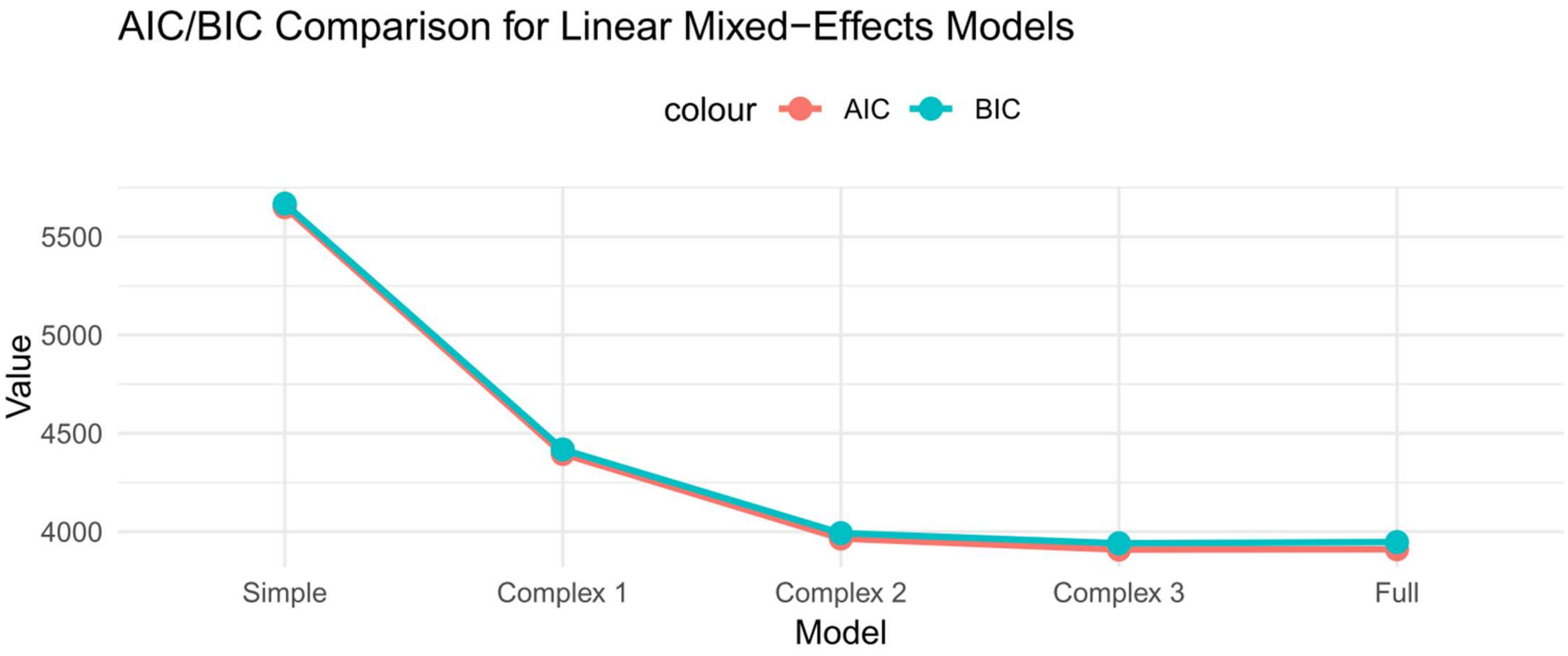

Supplement: Supplementary file 1 [file sensors-24-03343-s001.zip › Figure S4.pdf]
