# Supplementary figures and images for: Validation of Step Detection and Distance Calculation Algorithms for Soccer Performance Monitoring
Source: Sensors (Basel). 2024 May 23;24(11):3343. doi: 10.3390/s24113343 (PMC11174549; doi:10.3390/s24113343)

**Figure S1:** Q-Q plot of raw conditional residuals

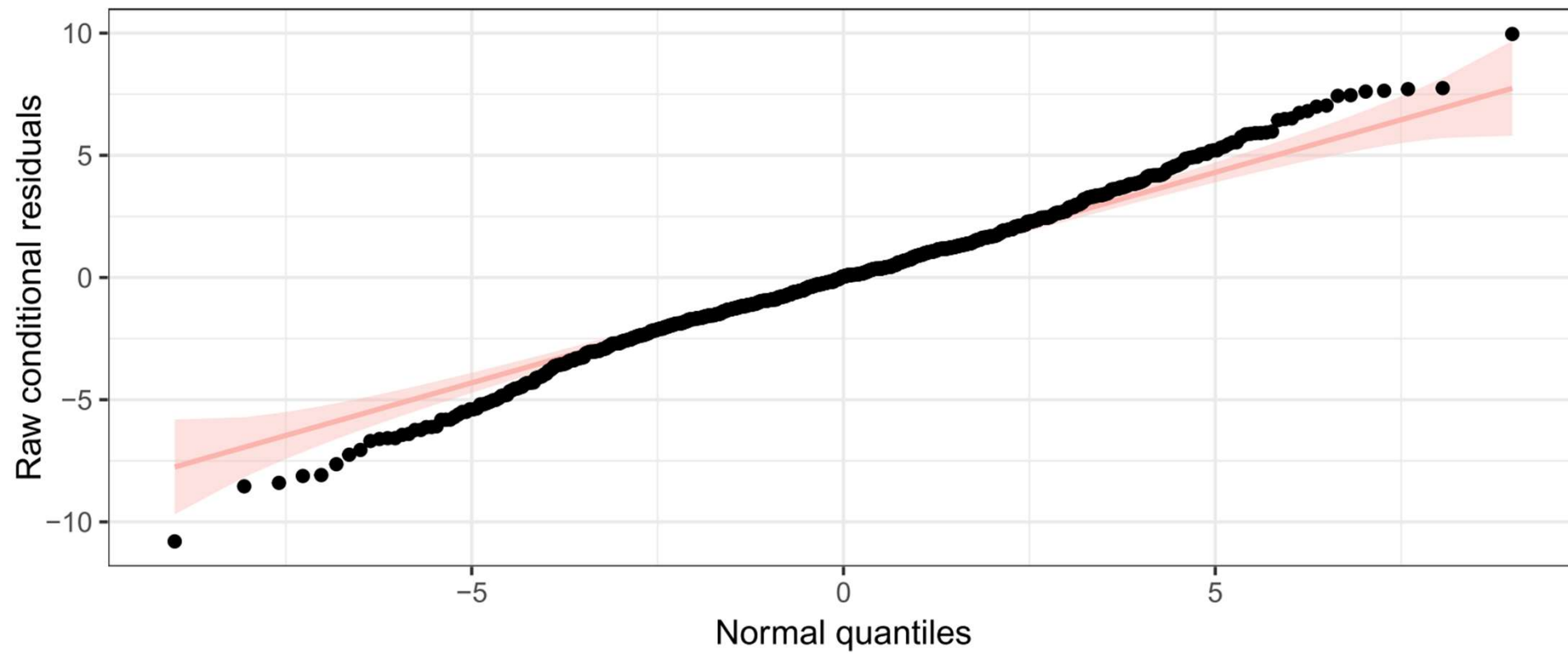

Supplement: Supplementary file 1 [file sensors-24-03343-s001.zip › Figure S1.pdf]

**Figure S2:** Plot of raw conditional residuals

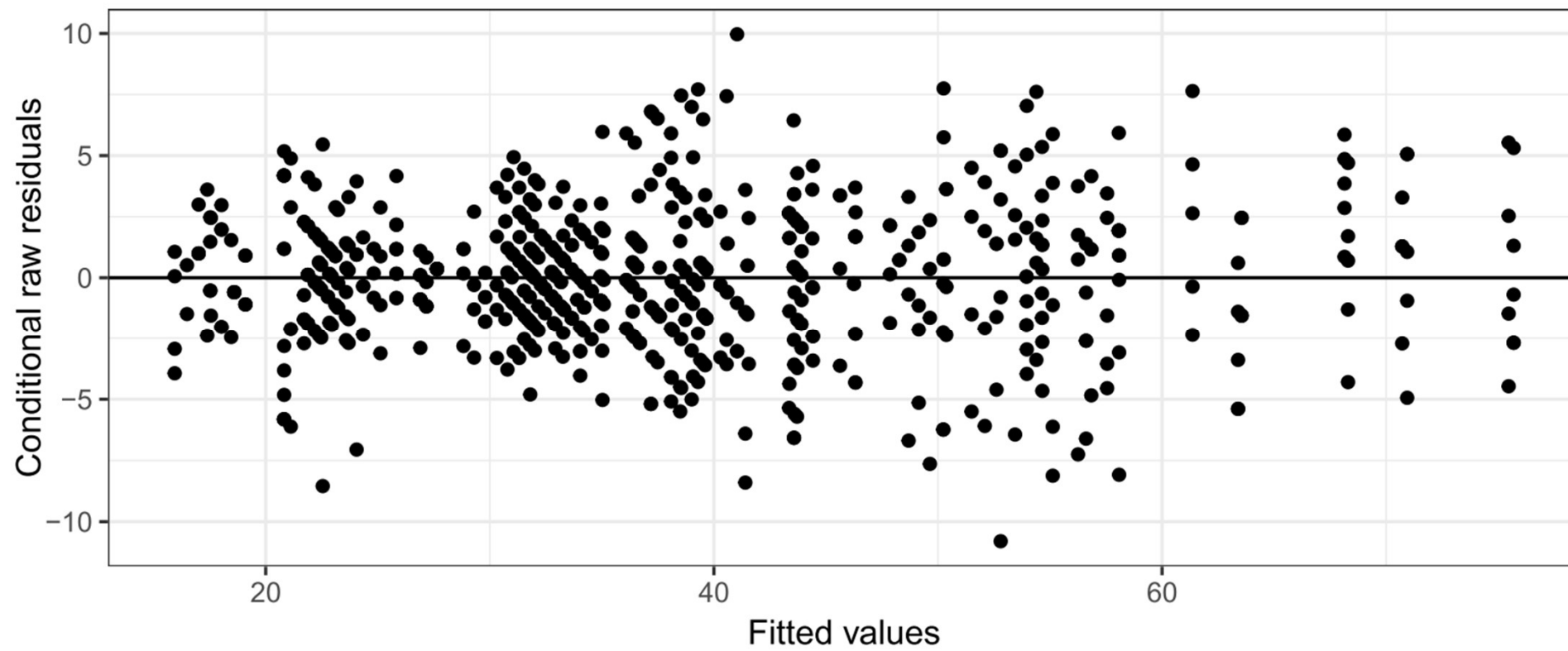

Supplement: Supplementary file 1 [file sensors-24-03343-s001.zip › Figure S2.pdf]

**Figure S3:** Plot of Pearson conditional residuals

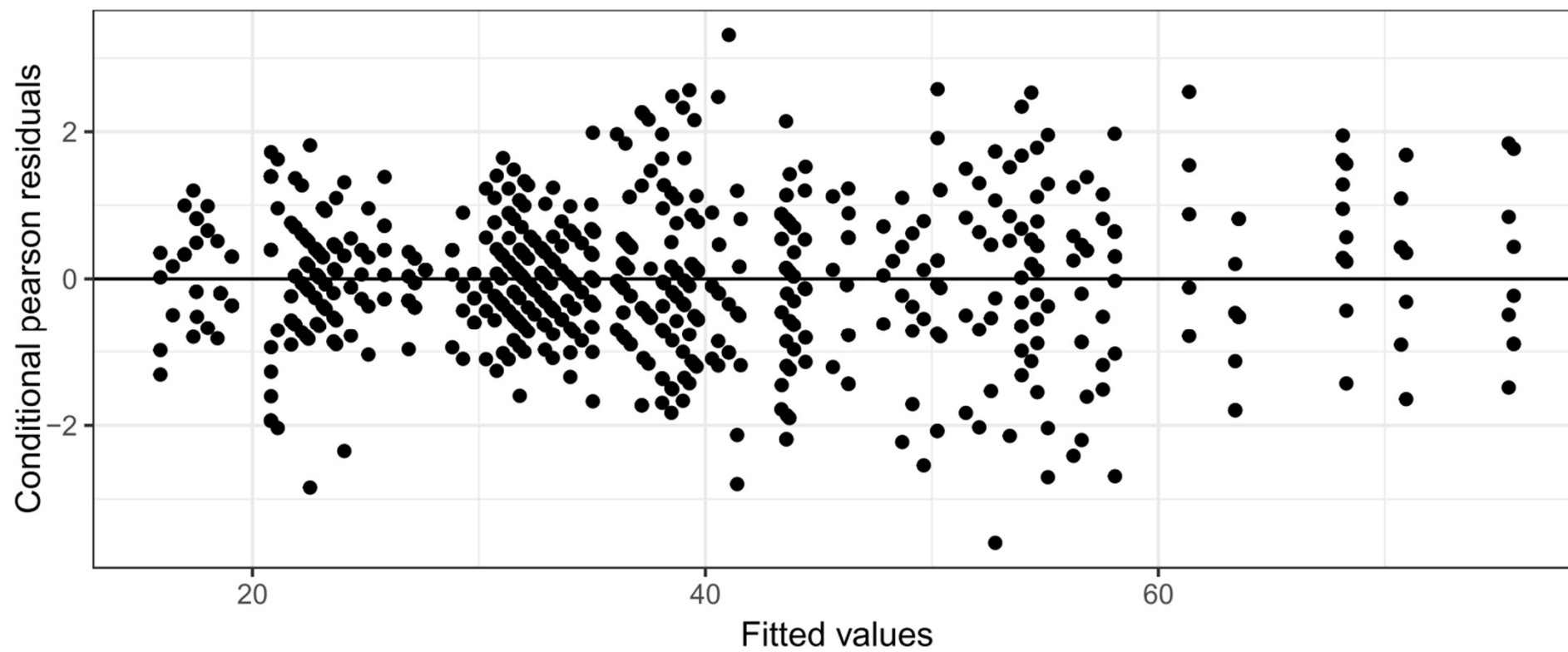

Supplement: Supplementary file 1 [file sensors-24-03343-s001.zip › Figure S3.pdf]
